# Effects of experimental nitrogen fertilization on planktonic metabolism and CO_2_ flux in a hypereutrophic hardwater lake

**DOI:** 10.1371/journal.pone.0188652

**Published:** 2017-12-12

**Authors:** Matthew J. Bogard, Kerri Finlay, Marley J. Waiser, Vijay P. Tumber, Derek B. Donald, Emma Wiik, Gavin L. Simpson, Paul A. del Giorgio, Peter R. Leavitt

**Affiliations:** 1 Department of Biology, University of Regina, Regina, Saskatchewan, Canada; 2 Environment Canada, Water Science and Technology Directorate, Saskatoon, Saskatchewan, Canada; 3 Groupe de recherche interuniversitaire en limnologie, Département des Sciences Biologiques, Université du Québec à Montréal, Montréal, Canada; University of Shiga Prefecture, JAPAN

## Abstract

Hardwater lakes are common in human-dominated regions of the world and often experience pollution due to agricultural and urban effluent inputs of inorganic and organic nitrogen (N). Although these lakes are landscape hotspots for CO_2_ exchange and food web carbon (C) cycling, the effect of N enrichment on hardwater lake food web functioning and C cycling patterns remains unclear. Specifically, it is unknown if different eutrophication scenarios (e.g., modest non point vs. extreme point sources) yield consistent effects on auto- and heterotrophic C cycling, or how biotic responses interact with the inorganic C system to shape responses of air-water CO_2_ exchange. To address this uncertainty, we induced large metabolic gradients in the plankton community of a hypereutrophic hardwater Canadian prairie lake by adding N as urea (the most widely applied agricultural fertilizer) at loading rates of 0, 1, 3, 8 or 18 mg N L^-1^ week^-1^ to 3240-L, *in-situ* mesocosms. Over three separate 21-day experiments, all treatments of N dramatically increased phytoplankton biomass and gross primary production (GPP) two- to six-fold, but the effects of N on autotrophs plateaued at ~3 mg N L^-1^. Conversely, heterotrophic metabolism increased linearly with N fertilization over the full treatment range. In nearly all cases, N enhanced net planktonic uptake of dissolved inorganic carbon (DIC), and increased the rate of CO_2_ influx, while planktonic heterotrophy and CO_2_ production only occurred in the highest N treatments late in each experiment, and even in these cases, enclosures continued to in-gas CO_2_. Chemical effects on CO_2_ through calcite precipitation were also observed, but similarly did not change the direction of net CO_2_ flux. Taken together, these results demonstrate that atmospheric exchange of CO_2_ in eutrophic hardwater lakes remains sensitive to increasing N loading and eutrophication, and that even modest levels of N pollution are capable of enhancing autotrophy and CO_2_ in-gassing in P-rich lake ecosystems.

## Introduction

Hardwater lakes and reservoirs exhibit some of the most extreme rates of atmospheric CO_2_ exchange of any ecosystem [1], yet the magnitude and direction of gas flux can vary dramatically in space and time [1–3], suggesting multiple regulatory mechanisms [4,5]. In general, hardwater lakes are alkaline (pH 8–11), rich in dissolved inorganic carbon (DIC) and nutrients, and highly productive [6,7], factors which favour rates of CO_2_ exchange (>200 mmol m^-2^ d^-1^) that greatly exceed those of other aquatic ecosystems [1,8,9]. Overall, the carbon (C) content of hardwater lakes is regulated by terrestrial subsidies of inorganic C rather than dissolved organic C (DOC) [10–12], with most dissolved C existing as bicarbonate (HCO_3_^-^) and carbonate (CO_3_^2-^) rather than free CO_2_, particularly when pH values exceed 8.5 [1,2]. They also exhibit intense heterotrophic metabolism [5,13] and temperature-dependent precipitation of CaCO_3_ [14,15] that can lead to supersaturation of CO_2_ even at elevated pH [1,11]. Taken together, high variation in both abiotic and metabolic processes can create large, and poorly understood, gradients in the magnitude and direction of CO_2_ flux from hardwater lakes [2].

Many of the world’s hardwater lakes are located in regions of intense agricultural management, urban growth, and eutrophication [1,2,16], with the influx of human-derived nutrients introducing another layer of mechanistic complexity to the regulation of lake C cycling and CO_2_ flux. In particular, application of N- and P-based fertilizers has increased ~500% since 1960 [17–19], and is most pronounced in regions where centuries of intensive agriculture have saturated soils with phosphorus (P) [20], increased terrestrial P export [21], and has caused the accumulation of soluble reactive P (SRP) in lakes despite elevated abundance of phytoplankton [22–24]. Unfortunately, up to 40% of N-based fertilizer can be exported to surface and ground waters, particularly when applied in association with cold temperatures, precipitation, irrigation, or chemical inhibitors of enzymatic decomposition [25–27]. N may also enter lakes through hydrologic transport of animal and human wastes, especially if microbial processing of effluent is limited [22,28–30]. Once in P-rich waters, both organic and inorganic dissolved N species can stimulate growth and toxicity of phytoplankton, as demonstrated by large-scale mesocosm experiments [24,31,32], whole-ecosystem fertilization ([33], but see [34]), and catchment-scale mass balance studies [22,23]. N enrichment may also enhance autotrophic metabolism and CO_2_ consumption, as rates of gross primary production (GPP) in hardwater systems are among the highest of all lake types [35]. However, little is known of how autotrophic responses balance with responses from the heterotrophic community to control net biotic CO_2_ fluxes.

Hardwater lakes commonly support rates of GPP in excess of respiration (R) during the ice-free season, as inferred from direct measurements at daily timescales [5,36], as well as organic carbon (OC) mass balance estimates over decades [10]. However, they are also often sites of intense microbial activity and elevated rates of R [10,13], and evidence suggests that pollution of eutrophic lakes with N may have differential effects on phytoplankton and bacteria. In the absence of fertilization, phytoplankton and periphyton are the main N sinks in surface waters [37–40]. Pelagic bacteria may acquire reduced N at <10% the rate of phytoplankton [41], and largely release simple DON compounds and NH_4_^+^ during decomposition of more complex organic substrates [42–44]. Moderate N inputs can elevate bioavailable N concentrations in surface waters [27,45] and selectively stimulate its consumption by prokaryotes [46,47], including cyanobacteria [44]. In contrast, extreme fertilization at levels characteristic of waste waters from urban and intensive-livestock sources (e.g., > 6 mg N L^-1^ week^-1^; [24,32]) can restrict phytoplankton growth due to lack of light or other factors [48–50], potentially resulting in elevated densities of heterotrophic bacterial growth [51–53]. Despite these generalities, little is known of the precise relationship between the magnitude of N pollution, relative effects on phytoplankton and bacterial assemblages (e.g., differential response, thresholds for effects), and net biotic effects on hardwater lake DIC dynamics.

Here, we conducted three 21-day mesocosm experiments to examine how a gradient of N pollution regimes regulate the magnitude and direction of auto-and heterotrophic planktonic metabolism, and DIC cycling in a hypereutrophic hardwater lake. We hypothesized that low to moderate levels of fertilization would favor planktonic autotrophy and increased CO_2_ uptake [24,32,54,55], but that more intense amendments characteristic of urban or agricultural effluent release may increase heterotrophic production [47,51,52] and CO_2_ saturation. Further, we anticipated that the importance of planktonic metabolism as a control of atmospheric CO_2_ exchange would vary during summer alongside physico-chemical controls on CaCO_3_ precipitation [1,11]. Better understanding of the relative effects of metabolism and lake-water chemistry as controls of atmospheric gas exchange has important implications for study of global CO_2_ cycles, because hardwater lakes strongly influence atmospheric CO_2_ exchange at regional [10,13,56] and global scales [1,2,9], yet are often impacted by urbanization and agricultural practises [16,22].

## Methods

### Study site and routine lake monitoring

We conducted three 21-day experiments in Wascana Lake (S1 Fig) located in the center of the City of Regina, Saskatchewan, Canada (50°26.17’N, 104°36.91’W). Necessary permits were obtained from the Wascana Center Authority, Saskatchewan Environment, and Transport Canada. Hypereutrophic Wascana Lake (0.5 km^2^) was created in the 1800s by the impoundment of Wascana Creek, but was deepened to 2 m in 1930s and to 7.5 m in 2004 [32,57]. At present, the lake lies within an urban park, receives drainage from a 1400 km^2^ agricultural catchment, and exhibits elevated but variable (mean ± SD) concentrations of total dissolved P (TDP) (299 ± 208 *μ*g P L^-1^) and soluble reactive phosphorus, SRP (200 ± 169 *μ*g P L^-1^). Because mass ratios of total dissolved N (TDN) to SRP are typically low (6.7 ± 6.6), phytoplankton communities are composed of N_2_-fixing (*Anabaena*, *Aphanizomenon*) and non-N_2_-fixing (*Microcystis*, *Planktothrix*) cyanobacteria [58], and are often growth-limited during summer by the supply of N [22,24,32]. During summer, zooplankton are composed mainly of small-bodied Cladocera and copepods [58], while large-bodied *Daphnia* are common only during the June clear-water phase [59].

The limnological characteristics of Wascana lake have been monitored during ice free (i.e., May-August, inclusive) periods for > 20 yr as part of the Qu’Appelle long term ecological research (LTER) program [13,57–59]. As in our earlier experimental work [24,32], we explored the patterns of Wascana lake ambient nutrient (TDN, TDP, SRP) and phytoplankton dynamics (biomass as chl *a* and nutrient bioassay incubation results) throughout the ice free period of 2009, to help place our experimental results into a broader context with respect to lake nutrient status and algal nutrient limitation. The Qu’Appelle LTER field and laboratory methods are consistent with experimental methods described below, and have been fully detailed in our earlier work [24,32].

### Mesocosm experiments

Fifteen mesocosms (2-m diameter, 1-m deep, ~3240 L) were attached to a floating wooden frame and deployed in a sheltered bay of Wascana Lake. To maximize our ability to interpret ecosystem dynamics, we replicated the experiment by conducting separate trials in July, August, and September 2009 [24,32]. As detailed in Finlay et al. [32], mesocosms were constructed from an opaque white poly-weave plastic held in shape with 3-cm thick plastic rings at the base and a ring of floatation material at the top of each enclosure. Mesocosms were open to the atmosphere, closed at the bottom, and did not include lake sediments. Each enclosure was passively filled by fully submerging to ~1.5 m depth, pulling it to the water surface, affixing floatation material, then filling to capacity by pumping unscreened water from 0.5 m depth. Minnow traps were placed in each enclosure to remove fish, but no attempt was made to modify biotic communities. Advantages and limitations of this experimental design have been discussed in Finlay et al. [32], but in general they accurately record planktonic processes [60] and atmospheric gas exchange [61].

Each experiment consisted of triplicate treatments of five rates of urea amendment (0, 1, 3, 8, and 18 mg N L^-1^ week^-1^). On days 0, 7, and 14, laboratory-grade urea was dissolved in 0.5 L of lake water in acid-washed polycarbonate bottles and mixed into mesocosms using a paddle. Urea was added since we knew from previous results that it stimulates phytoplankton growth in summer under N-limited conditions [24,32]. Sampling was conducted immediately before urea addition (10:00–12:00 h), except for day 0 during August and September trials when sampling followed urea addition. On each sampling date (days 0, 4, 7, 14, and 21), we measured water temperature, conductivity, and oxygen concentration using either a YSI model 85 (July) or YSI 600XL probe with 650 MDS monitor (August, September). In addition, surface pH was determined using a calibrated handheld Oakton pH meter, while water transparency was estimated using a 20-cm Secchi disk. Due to equipment failure, pH (day 21) was not measured on all dates during the July experiment. Water samples for chemical and biological analyses were collected at 0.5-m depth with a 2.2-L Van Dorn water bottle, screened through a 243-*μ*m Nitex mesh to remove large invertebrates, and transported to the lab in 10-L carboys.

### Chemical analyses

Water chemistry was determined for samples filtered through 0.45-*μ*m pore cellulose membrane filters and stored at 4°C. Concentrations of nitrate + nitrite (NO_3_^-^ hereafter), ammonium + ammonia (NH_4_^+^ hereafter), SRP (as orthophosphate), and total dissolved phosphorus (TDP) were determined following standard methods at the University of Alberta Water Chemistry Laboratory [62]. Analysis of dissolved inorganic carbon (DIC) and dissolved organic carbon (DOC) concentrations followed standard Environment Canada [63] procedures using a Shimadzu model 5000A total carbon analyzer.

### Plankton abundance and productivity

Whole-water samples for analysis of algal abundance and productivity were collected from 0.5-m depth using a 2.2-L Van Dorn water bottle. Phytoplankton abundance was estimated by concentrating particulate organic matter (POM) onto duplicate GF/C filters (1.2-*μ*m pore), extracting pigments using pure acetone, and determining Chl *a* concentrations (*μ*g L^-1^) using standard spectrophotometric equations [64] employed for Wascana Lake since 1996 [57]. Gross primary productivity (GPP) was measured in situ following slightly modified methods of Waiser and Robarts [65]. Here aliquots of pre-screened water (as above) from each mesocosm were added to 1 light and 1 dark bottle, amended with 400 *μ*L of NaH^14^CO_3_ (0.26 MBq), and incubated at 0.5 m for 3 h (10:00–13:00 h). Incubations were ended by placing the bottles in a light-proof case until filtration onto 0.45-*μ*m pore cellulose-nitrate filters, and acidification overnight with 500 *μ*L of 1N HCl. Filters were dissolved in 10 ml of Filter Count scintillation fluor solution and activity determined using a Canberra Packard 1900 CA scintillation counter. Daily rates of GPP (mg C m^-3^ d^-1^) were determined by multiplying hourly rates by 10 assuming a 10-h sunlight period [65]. Triplicate analyses were conducted for each mesocosm on day 4 of each experiment. Within individual enclosures, the range in estimates of GPP on day 4 were well constrained (mean of S.D. in each bag = 90.8, 106.2, and 90.7 mg C m^-3^ d^-1^ in July, August, and September respectively). Enclosure-level variability was 4 to 7-fold smaller than at the treatment-level (mean of S.D. among bags at each treatment level = 705, 428, 577 mg C m^-3^ d^-1^, respectively), and nearly 2 orders of magnitude smaller than the range in rates of GPP in all data across treatments (> 7000 mg C m^-3^ d^-1^ in each experiment). Given the extremely small variability in GPP within enclosures relative to treatment and inter-treatment level variability, we collected only 1 sample per bag on all other days in each experiment.

Bacterial abundance in whole water samples was measured using flow cytometry following del Giorgio et al. [66]. Lugol’s preservative (0.5 ml) was added to 10-mL samples of whole water, and samples were stored in the dark at 4°C until analysis, at which time they were de-stained with sodium thiosulphate [65]. Bacterial productivity (BP) was estimated in triplicate for each mesocosm by adding 15 nM methyl-^3^H thymidine (TdR) to 10 mL of gently sonicated, screened (as above) lake water in 20-mL glass vials [65]. Killed control samples received 500 *μ*L of formaldehyde and 500 *μ*L of 5 N NaOH. Samples were incubated for 30 min in Wascana Lake adjacent to the enclosures and were ended by addition of NaOH and formaldehyde (as above). Samples were transported to the laboratory at 0°C, DNA extracted according to Robarts and Wicks [67], and radioactivity estimated using a Canberra Packard 1900 CA scintillation counter. Incorporation of TdR into DNA was estimated from sample activities and by converting uptake to cell production assuming 1 mole TdR = 2.0 × 10^18^ bacterial cells produced and that each cell contained 20 fg C [68]. Total bacterial C consumption (BCC; productivity + respiration) was estimated by assuming a bacterial growth efficiency of 35%, which is common for eutrophic ecosystems [69]. Rationale for use of this conversion factor in eutrophic prairie ecosystems is provided by Waiser and Robarts [65]. Daily BCC was estimated from hourly determinations by multiplying by 24 [65]. Ratios of GPP:BCC were used as an approximate indicator (in conjunction with other estimates, such as DO percent saturation) to assess the planktonic metabolic balance in enclosures.

### Quantifying inorganic C dynamics

Dissolved CO_2_ concentrations were calculated for each sampling date in all mesocosms using DIC concentrations and surface-water pH values following Finlay et al. [10,13], with correction for ionic strength and water temperature [70]. Partial pressure of CO_2_ (μatm) was estimated using Henry’s Law constant and accounted for changes in temperature [71]. As our enclosures were all > pH 8 (see below), it is extremely unlikely that our calculations would have seen any error associated with calculated *p*CO_2_, as is common in more acidic, DOC-rich softwater systems. Chemically enhanced CO_2_ flux (mg C m^-2^ d^-1^) was calculated for each sampling date following the boundary-layer equations presented in Cole and Caraco [72]:

$$CO2\;flux\;=\;\alpha \;\times \;k_{CO2}\;\times \;\Delta {CO}_{2}$$

where ΔCO_2_ is the observed concentration of dissolved CO_2_ in the surface water minus the concentration of CO_2_ at equilibrium with the atmosphere (mg C L^-1^), α is the chemical enhancement of CO_2_ flux at high pH [73] calculated using the equations of Wanninkhof and Knox [74]; and *k*_CO2_ is piston velocity (i.e., gas exchange coefficient in m d^-1^) determined from equation 5 in Cole and Caraco [72] relating *k*_CO2_ to wind speed, accounting for temperature [75]. Here, positive values of CO_2_ flux represent net influx of atmospheric CO_2_ into the mesocosm. Atmospheric *p*CO_2_ was assumed to be 385 μatm for the duration of the study. As the objective of this study was to isolate the biological and chemical effects of fertilization with urea on CO_2_ flux, we have used the long-term average wind speed of 2.8 m s^-1^ for Wascana Lake to calculate *k*_CO2_ throughout the experiment following Finlay et al. [13]. Bi-weekly wind speed measurements recorded in 2009 on Wascana Lake (mean = 2.9; SD = 3.4 m s^-1^) were consistent with long term averages used in this study. We are confident that our estimates of *k*_CO2_ sufficiently captured the patterns in air water gas flux, and that any errors linked to our methodology would have had little impact on our general results and broader conclusions: Past comparison [42] of empirical gas flux measurements have shown that the wind-based approach used here effectively characterizes the mean daily-scale air-water gas exchange characteristics of mesocosms deployed in small lakes. In addition, the large chemical gradients in pH and *p*CO_2_ generated by N amendments (see below) far exceed any potential variability linked to our estimates of *k*_CO2_.

### Data analysis

Repeated-measures analysis of variance (RM-ANOVA) was used to test the effects of urea fertilization on physical, chemical and biological parameters. This approach has been described in detail by Finlay et al. [32]. Where possible, least-squares regression analysis was used to quantify the linear and non-linear relationships between urea loading rates and mean (day 7–21) parameters. We tested a suite of linear and non-linear models on each relationship to best explain variance in the data. Model fit was evaluated using Akaike’s Information Criterion adjusted for small sample sizes (AIC_c_) [76]. RM-ANOVAs were performed using SPSS v. 16, while regression models were generated using Sigma Plot v. 12. Data underlying these analyses are provided as supporting information (S1 Dataset).

## Results

### Initial conditions

**Fig 1:**
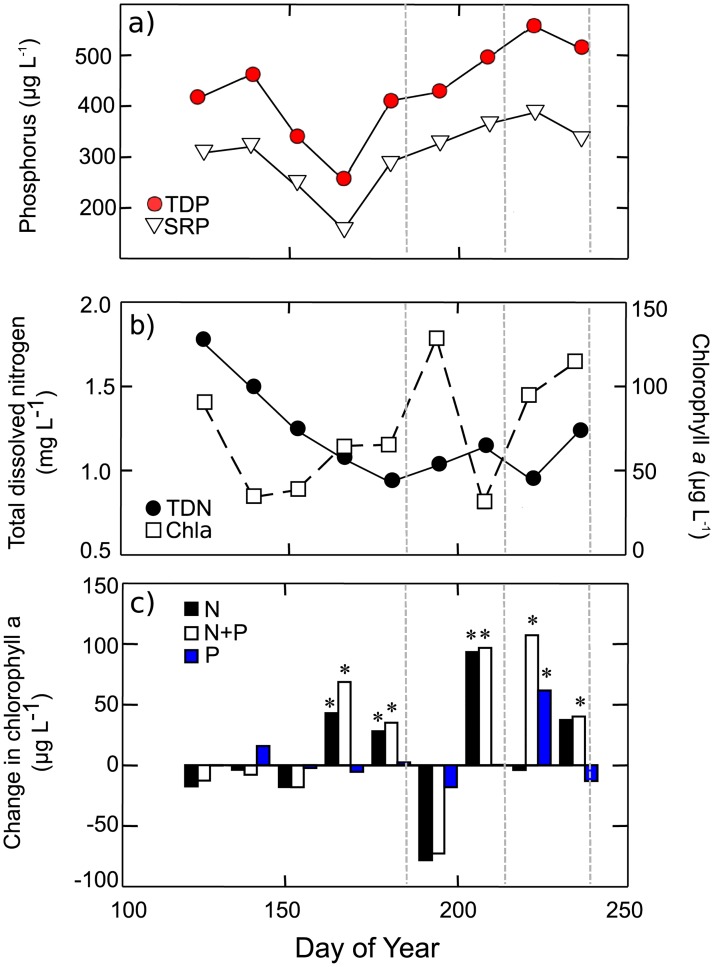
Seasonal limnological trends in Wascana Lake, Saskatchewan May–August 2009. (a) total dissolved (TDP) and soluble reactive phosphorus (SRP) concentrations, (b) total dissolved nitrogen (TDN) concentration and phytoplankton biomass (as Chl *a*), and (c) final concentrations of Chl *a* (fertilized treatment minus control) after 72-h bottle bioassay incubations of Wascana Lake water receiving growth-saturating concentrations of NH_4_ (N), PO_4_^3-^ (P), or both N and P (N+P). Analysis of variance with Tukey’s post hoc tests identified statistically significant (asterisk) phytoplankton biomass response (*p* < 0.05) relative to control bottles. Vertical dashed grey lines show the start dates of the monthly mesocosm experiments.

Routine monitoring of Wascana Lake during 2009 revealed little difference in initial nutrient conditions for July, August and September experiments (Fig 1). Both P (400–500 μg TDP L^-1^ and 280–400 μg SRP L^-1^) and N (0.9–1.4 mg TDN L^-1^) were elevated during July-September (Fig 1a and 1b), consistent with previously reported long-term monitoring observations [57]. Mean mass ratios of TDN:SRP were low (2.7–3.5), and nutrient enrichment bioassays demonstrated that N supply limited instantaneous growth of phytoplankton through much of the summer (Fig 1c). As in most regional lakes, TDN was composed mainly (~80%) of non-urea DON [45] whereas DIC levels were elevated (40–50 mg C L^-1^) throughout the summer (S3b Fig). Although depth-integrated phytoplankton abundance fluctuated between 50 and 125 μg Chl *a* L^-1^ throughout the summer (Fig 1b), Secchi disc transparency was low (0.4–0.6 m) at the onset of each experiment (S4e Fig). In contrast, initial pH values declined from ~10.5 in July to ~9.0 in August and September (S3a Fig).

### Phytoplankton and bacterial response to N

**Fig 2:**
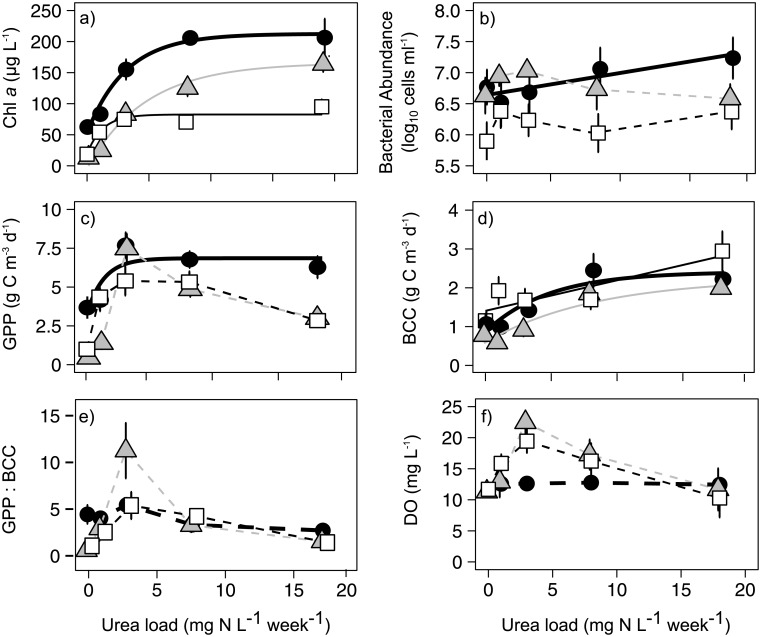
Effects of urea fertilization rate (mg N L^-1^ week^-1^) on mean planktonic parameters. Results averaged for days 7–21. Response variables include (a) phytoplankton biomass (as Chl *a*), (b) bacterial density, (c) gross primary production (GPP), (d) bacterial C consumption (BCC; productivity + respiration), (e) the approximate metabolic balance of plankton communities, measured as GPP: BCC, and (f) dissolved oxygen concentration (DO). Data in each panel includes July (black circles and thick black lines), August (grey triangles and grey lines), and September (white squares and thin black lines) experiments. Solid lines indicate best-fit regression models detailed in Table 1, dashed lines indicate direction of change for trial in which statistically-significant regression models could not be fit. Error bars = ± 1 S.E, and *n* = 9.

Mean (days 7–21) phytoplankton abundance (Fig 2a) and GPP (Fig 2b) increased as a function of N input to a plateau at 3–5 mg N L^-1^ week^-1^, beyond which algal growth was either sustained or declined. Least squares regression models selected using AICc (Table 1) suggested that phytoplankton growth was best described using two- or three-term exponential models of non-linear increase to plateau values, although sharp declines in GPP in mesocosms treated with >3 mg N L^-1^ week^-1^ prevented the fitting of any regression models to GPP data during August and September experiments (Fig 2b, Table 1). Phytoplankton biomass (as Chl *a*) increased three- to six-fold (*P*_treatment_ < 0.001) above the initial mesocosm concentrations of 25–50 μg Chl L^-1^ (S1 Table), with the magnitude of response generally increasing as a function of the rate of urea amendment (Fig 2a). Similarly, urea amendments increased gross primary productivity (GPP) by up to three-fold, with the greatest response usually in mesocosms receiving 3 mg N L^-1^ week^-1^ (Fig 2c). In most cases, phytoplankton abundance and GPP increased within 4–7 days to plateaus of ~80–200 μg Chl *a* L^-1^ and ~6–8 g C m^-3^ day^-1^, respectively (S2a and S2b Fig; S1 Table). Enclosures receiving N mostly underwent 0.3–0.4 m declines in water transparency relative to initial conditions, while control mesocosms became substantially (~0.5 m) more transparent (S4e Fig).

**Table 1:** Model fits describing N effect on mesocosm plankton dynamics. Least squares regression analysis (*n* = 15) of phytoplankton and bacterial abundance and production (y) as functions of urea load (x). Models were selected using Akaike information criterion corrected for small sample sizes (AIC_c_), and ranked based on AIC_c_ score, with best-fitting models in bold. Models with no explanatory power (i.e. r^2^ = 0) are omitted. See Fig 2 for graphical representation of best-fit models.

Experiment	Model	RSS	AICc	r^2^
Chlorophyll *a* (mg L^-1^)				
July	**y = 55.02+157.43(1-1e**^**(-0.31x)**^**)**	**338.2**	**61.9**	**0.98**
	y = 207.46(1-1e^(-0.48x)^)	3941.2	93.4	0.78
	y = 97.72+7.48x	5947.3	99.6	0.67
August	**y = 165.74(1-1e**^**(-0.20x)**^**)**	**210.7**	**49.5**	**0.98**
	y = 8.94+160.64(1-1e^(-0.17x)^)	319.2	61.1	0.99
	y = 33.40+8.10x	2313.7	85.4	0.86
September	**y = 19.17+63.43(1-1e**^**(-0.76x)**^**)**	**305.7**	**60.4**	**0.91**
	y = 81.60(1-1e^(-1.04x)^)	670.4	66.8	0.79
	y = 44.07+3.09x	1176.3	75.3	0.64
GPP (mg C m^-3^ day^-1^)				
July	**y = 3365.66+3485.31(1-1e**^**(-0.69x)**^**)**	**2925608**	**197.9**	**0.73**
	y = 5085.02+105.25x	9438501.37	210.1	0.203
Bacterial Abundance (cells ml^-1^)				
July	**y = 12233695+26453509(1-1e**^**(-0.22x)**^**)**	**1.809E+14**	**432.4**	**0.96**
	y = 17667687+1295526x	1.293E+14	456.6	0.74
	y = 37103523(1-1e^(-0.42x)^)	2.110E+14	463.9	0.57
BCC (mg C m^-3^ day^-1^)				
July	**y = 884.69+1524.85(1-1e**^**(-0.21x)**^**)**	**225275**	**159.5**	**0.87**
	y = 1190.77+73.09x	622761	169.3	0.65
	y = 2330.29(1-1e^(-0.41x)^)	1287189	180.2	0.28
August	**y = 572.25+1671.52(1-1e**^**(-0.12x)**^**)**	**163337**	**154.6**	**0.9**
	y = 749.41+79.11x	316880	159.2	0.81
	y = 2052.71(1-1e^(-0.24x)^)	658758	170.2	0.61
September	**y = 1406.17+78.72x**	**394574**	**162.5**	**0.77**

Mean bacterial abundance increased as a function of N amendment in July, but changed little in other months (Fig 2b). In all months, BCC increased as a function of N additions (Fig 2d), with shallow non-linear models providing the best fit to the data during July and August, and a linear model best describing changes in the September experiment (Table 1). In contrast to rapid (<7 days) phytoplankton responses, bacterial densities showed little response to added urea (S2c Fig). Treatment with ≥ 8 mg N L^-1^ week^-1^ significantly (*P*_treatment_ <0.01) increased bacterial densities two-fold during July trials, but not during August or September experiments (S1 Table). On the other hand, BCC increased two- to five-fold (*P*_treatment_ < 0.01, *P*_interaction_ < 0.001) relative to initial rates in all months, with particularly elevated BCC observed in trials receiving 18 mg N L^-1^ week^-1^ (S2d Fig; S1 Table).

### Effects of N on the metabolic balance of plankton

The differential responses of phytoplankton and bacterial communities to added N caused substantial changes in the metabolic balance of pelagic communities (as GPP: BCC), particularly during August and September experiments (Fig 2e). During the latter two months, mean ratios of GPP:BCC increased 5-10-fold to maxima characteristic of highly autotrophic conditions (GPP > 5 × BCC) in trials receiving 3 mg N L^-1^ week^-1^, but declined to near-initial ratios in the most heavily amended treatments. In the July experiment, the greatest shift to net autotrophy also occurred in the 3 mg N L^-1^ week^-1^ treatment, though the GPP: BCC ratio was ~21% lower than that observed during other months (Fig 2e).

Consistent with overall patterns in GPP:BCC, daytime DO concentrations increased as a function of N addition, to a maximum of >20 mg O_2_ L^-1^ (>200% saturation) in the 3 mg N L^-1^ week^-1^ treatments, whereas DO declined to baseline concentrations in the more heavily fertilized treatments (Fig 2f). All levels of N fertilization stimulated photosynthetic activity sufficiently to increase O_2_ concentration from initial values of ~10–15 mg L^-1^ (110–170% saturation) to supersaturated concentrations of 20–30 mg L^-1^ (mean 235 ± 20% saturation) by day 4, although O_2_ concentrations declined beyond that time in all experiments (S2f Fig). Reductions in O_2_ were particularly marked in enclosures receiving 8 or 18 mg N L^-1^ (S1 Table) with O_2_ levels being reduced < 5 mg L^-1^ by day 21 (mean 45 ± 31% saturation; S2f Fig), and as low as 0.8 mg L^-1^ in one enclosure of the highest N treatment.

### Effects of N on inorganic C cycling

**Fig 3:**
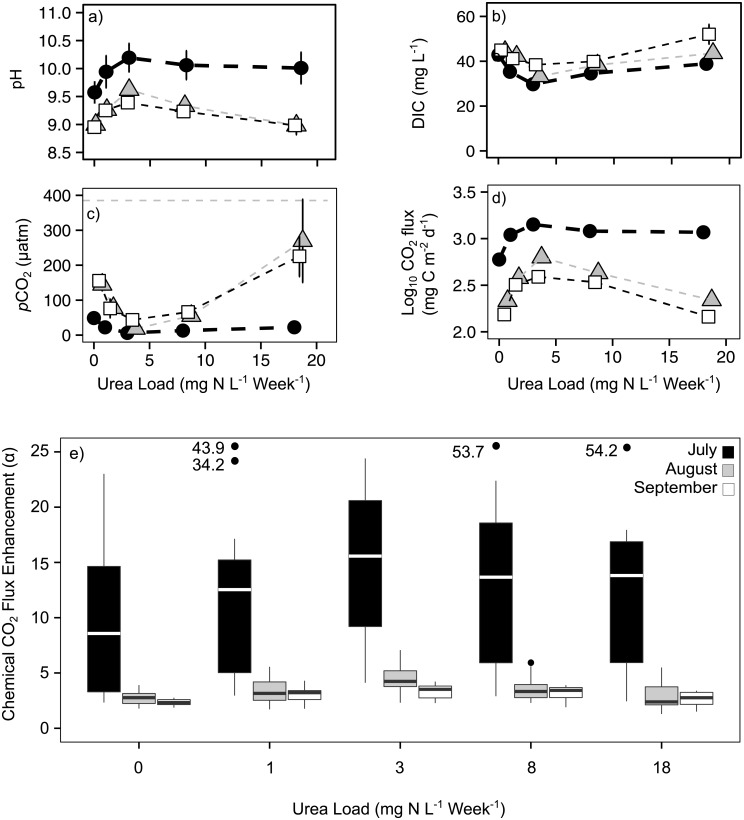
Effect of N influx rate (mg N L^-1^ week^-1^) on carbon cycling and chemically-enhanced CO_2_ flux in mesocosms. Data from days 7–21 were averaged for (a) pH, (b) DIC, (c) partial pressure of CO_2_ (*p*CO_2_) and for (d) the rate of air-water flux of CO_2_ (positive values represent influx). In July, averages of days 7–14 were used for pH, *p*CO_2_ and emissions rates. In all cases, symbols are consistent with Fig 2. Error bars = ± 1 S.E, and *n* = 9 (*n* = 6 in July). (e) The calculated role of chemical enhancement of CO_2_ fluxes for each month, by treatment level. Boxes depict the median (central line), as well as 1^st^ and 3^rd^ quartile (box limits). The horizontal dashed grey line in panel c depicts atmospheric *p*CO_2_ (385 μatm).

In each experiment, N additions caused substantial changes in pH, DIC and *p*CO_2_ within fertilized mesocosms (Fig 3a–3c). The lake naturally underwent seasonal changes in *p*CO_2_ and thus pH, resulting in the lowest starting *p*CO_2_ (and consequently highest pH) occurring on day 0 of the July experiment, relative to day 0 of August and September experiments (S3a and S3c Fig). All levels of N addition initially increased pH relative to controls in each experiment, followed by declines in pH beyond day 4–7 (S3a Fig). Overall, mesocosms receiving 1–3 mg N L^-1^ week^-1^ exhibited lower *p*CO_2_ and DIC content relative to both controls and heavily amended mesocosms (Fig 3b and 3c), resulting in a non-linear relationship between *p*CO_2_ and N influx that continued until the end of each experiment.

As July experiments began with much lower day 0 *p*CO_2_ (and thus higher pH, as mentioned above) than August and September (S3d Fig), this led to persistently greater rates of CO_2_ in-gassing in July for each treatment, as compared to later months (Fig 3d). Within each experiment, rates of CO_2_ influx were greatest for mesocosms receiving 1–8 mg N L^-1^ week^-1^ (*P*_treatment_ & *P*_interaction_ < 0.001 in August and September), largely because of the intense autotrophic growth and drawdown of *p*CO_2_ in the surface waters (Fig 3c; S1 Table). Within most experiments, CO_2_ influxes increased rapidly following fertilization with urea, then declined beyond day 7, although net CO_2_ efflux was observed only on the last day of late summer experiments receiving 18 mg N L^-1^ week^-1^ (S3d Fig). Overall, chemical enhancement of CO_2_ flux was two- to 10-fold greater in July than in later months (Fig 3e).

We also observed evidence that CaCO_3_ precipitation likely influenced CO_2_ dynamics in all treatments in July, and possibly in the 3 and 8 mg N L^-1^ week^-1^ treatments in August: In July, the initial pH was exceptionally high (average 10.48 ± 0.23; S3a Fig) which likely triggered precipitation of CaCO_3_. This pattern appears to be irrespective of fertilization treatments and rates of photosynthesis, as all mesocosms experienced DIC concentrations dropping from an average of 44.0 (± 1.0) to 33.7 (± 6.8) μg L^-1^ (S3b Fig) and a decline in pH by over an entire unit (average 9.36 ± 0.25 after 2 weeks incubation; S3a Fig). Similarly, we observed a smaller loss of DIC in the 3 and 8 mg N L^-1^ week^-1^ treatments in August (S3b Fig) that were accompanied by 0.25 to 0.5 pH unit declines (S3a Fig). These events, however, were not sufficient to shift the net direction of CO_2_ flux, and all treatments consistently in-gassed CO_2_ during these precipitation events (Fig 3d, S3d Fig).

## Discussion

Human population growth, increased fertilization for N-rich food stuffs, and disproportionate rates of urban development are expected to double N influx to the environment within 40 years [19,26], which as suggested here, have major ramifications for aquatic ecosystem functioning. Experimental fertilization of P-rich mesocosm waters with near-ambient levels of N (1–3 mg N L^-1^ week^-1^) increased Chl *a*, GPP and chemically-enhanced CO_2_ influx by up to 600% at the scale of days-to-weeks (Figs 2 and 3). Higher levels of fertilization (>8 mg N L^-1^ week^-1^) selectively increased bacterial C consumption, net planktonic CO_2_ production and, CO_2_ outgassing on the final day of August and September experiments (S3d Fig). Though calcite precipitation events in July lowered pH by > 1 unit due to the generation of CO_2_, it had no effect on the direction (i.e., in-flux) of CO_2_ throughout these events. Overall, our findings from this series of mesocosm experiments demonstrate that CO_2_ exchange between the atmosphere and hardwater ecosystems is highly sensitive to even modest increases in N pollution, thereby underscoring the intricate connection between the biogeochemical cycles of both inorganic C and N in continental hardwater lakes.

### Planktonic responses to N loading

Fertilization of P-rich eutrophic waters with N resulted in two- to six-fold increases in phytoplankton abundance (Fig 2a) and productivity (Fig 2b) consistent with findings from other N fertilization experiments in laboratory [44,77], mesocosm [31–33], whole ecosystem [33], and catchment-scale settings [22,23]. Strong phytoplankton responses were also consistent with thresholds for N effects identified by Donald et al. [24] in which stimulation of phytoplankton by N is restricted to waters in which SRP is > 50 μg P L^-1^ and TDN: SRP is < 20: 1, by mass. Additionally, our new analyses demonstrated that stimulation of phytoplankton by N occurred at levels much lower than previously identified (1 mg N L^-1^) and which were similar to ambient concentrations of dissolved N in other hardwater lakes [6,7,45].

Thresholds in both the magnitude (Fig 2a and 2c) and duration (S2a and S2b Fig) of phytoplankton response to a gradient of N fertilization suggest that planktonic assemblages may exhibit a finite capacity to assimilate N. The temporal responses of phytoplankton seen in each of our experiments are consistent with previous experiments in Wascana Lake, where the maximal phytoplankton response to N amendments occurred within ~7 days, after which there was little additional accumulation of biomass either as Chl *a* (S2a Fig) or cellular biovolume [32,78]. Several mechanisms may act in concert to limit the magnitude of phytoplankton response to added N. First, biological assimilation of SRP (S4c Fig), combined with elevated N: P ratios following urea amendments may have induced P-limitation of phytoplankton growth in the heaviest N-addition treatments [79]. As noted previously, effects of N pollution on water quality appear to be restricted to P-rich environments with low N: P mass ratios [24,73]. Second, progressive reduction in water-column transparency (S4e Fig) may have induced light limitation of phytoplankton production, as seen in other highly eutrophic systems [49,50]. Third, rapid increases in biomass of phytoplankton within mesocosms may have induced micronutrient limitation of growth, including elements that act as cofactors for enzymes involved with active uptake of N (Fe, Mo) [80] or urea decomposition (Ni) [41]. Though accumulation of inorganic N can suppress growth of phytoplankton via toxic effects in some circumstances [81], we do not favor this explanation as toxic effects of NH_4_^+^ are most pronounced for diatoms, taxa which are uncommon in later summer in Wascana Lakes [57,78]. Similarly, we infer that competition for nutrients with bacteria did not inhibit phytoplankton response to added urea [81] because primary production was usually much greater than that of bacteria (Fig 2e), and reduced N (both NH_4_^+^ and urea) was still available in the water column later in the experiments for the higher N-load treatments, when bacterial growth was enhanced (data not shown).

Consistent with findings from other experiments (e.g., [82]), initial bacterial response to added N was muted relative to that of phytoplankton, with maxima of cell density and productivity occurring only at the end of experiments (S2c and S2d Fig). Several lines of evidence suggest that direct assimilation of urea-derived N and C into bacterial biomass was relatively unimportant in these experiments. First, the mean bacterial abundance and productivity increased only two-fold in response to an 18-fold gradient of urea supply (Fig 2b and 2d). Second, bacteria already had sufficient access to DOM prior to urea additions, as Wascana Lake DOC already exceeded 10 mg C L^-1^ (S4d Fig), and DON comprised >80% of TDN throughout the summer [45]. Similarly, weak correlations between GPP and BCC (*r*^2^ = 0.02–0.20, *P* <0.20) suggest that there was little indirect effect of N on bacterial growth via release of labile exudates from phytoplankton [83,84], although we recognize that decomposition of phytoplankton over longer periods may have supported the intense bacterial growth observed by day 21 of experiments (S2d Fig). Instead, we hypothesize that the cycling of inorganic N by chemolithotrophs may represent a major indirect effect of the N additions on the heterotrophic bacterial community, although this hypothesis is largely speculative and further research is needed to confirm this expectation (S1 Text).

### N-driven shifts in the metabolic balance of plankton

Due to the differential response of phytoplankton and heterotrophic bacteria, the metabolic balance of planktonic communities exhibited a non-linear response to increased nutrient additions. Moderate enrichment at 1–3 mg N L^-1^ week^-1^ favored autotrophic conditions and DIC uptake in mesocosms, as ratios of GPP: BCC increased to nearly 5: 1 (Fig 2e), extreme super-saturation of oxygen to >200% saturation (Fig 2f), elevated pH (Fig 3a), and 20–40% declines in DIC concentration (Fig 3b). These patterns are consistent with the effects of elevated photosynthesis in alkaline waters, including enhanced HCO_3_^-^ uptake and concomitant pH increase [2, 54]. Further, these findings are congruent with whole-lake mass balance studies that show P-rich Qu’Appelle lakes are autotrophic [10] and are eutrophied further by the influx of dissolved N [22]. Interestingly, stimulation of GPP occurred at N concentrations (1–3 mg N L^-1^) similar to TDN values recorded in most regional lakes [45]. This suggests that even modest levels of pollution with urea, and likely other forms of reduced N [34], are capable of enhancing autotrophy in P-rich lake ecosystems.

Addition of N at rates characteristic of the influx of untreated livestock wastes and primary-treated urban effluent [51,52,85] coincided with declines in GPP: BCC down to near unity due to both increased bacterial metabolism and declines in autotrophic production (Fig 2e). In particular, bacterial densities (up to 5 × 10^7^ cells mL^-1^) and C cycling (up to 5 g C m^-3^ day^-1^) (S2c and S2d Fig) greatly exceeded the range observed in most other lakes [86]. Increased heterotrophic metabolism at the community level (i.e., including all plankton, not just bacteria) eventually favored net biotic CO_2_ production, as inferred from the depleted O_2_ concentrations that created hypoxic conditions in mesocosms receiving ≥ 8 mg N L^-1^ week^-1^ (S2f Fig). In these heavily-amended enclosures, the accumulation of NO_3_^-^ (data not shown) may have supported extreme metabolic rates of denitrifying bacteria which favoured a more heterotrophic state (S1 Text).

### Roles of CaCO_3_ precipitation and metabolism on CO_2_ flux

Any CaCO_3_ precipitation events that likely occurred during the experiments (particularly in July) did not add enough CO_2_ to the enclosures to overcome the effects of N additions on net autotrophy, planktonic CO_2_ consumption, and CO_2_ in-gassing. Additions of N that stimulated autotrophic growth (S2e and S2f Fig) and pH increases to ~11 (S3a Fig) in the first 4 days of the July experiment appeared to induce major DIC losses during a precipitation event in the mesocosms. Without corresponding Ca^2+^ data for the enclosures, we inferred this precipitation event during days 4–14 from the ~25% drop in DIC concentrations and simultaneous drop in pH (S3a–S3d Fig). This event corresponded to a *p*CO_2_ increase in the mesocosms and reduction in the rates of CO_2_ influx (S3c and S3d Fig). Unlike observations in other studies [14,87], this large contribution of CO_2_ to enclosures still did not result in outgassing (Fig 3d; S3d Fig), because autotrophic growth remained high and exceeded heterotrophy for the duration of July (Fig 2e; S2f Fig). Unfortunately, the failure of the pH meter prior to the third week of sampling in July precludes us from observing the longer-term effects of this precipitation event on *p*CO_2_ dynamics, but DIC trends appeared to stabilize after day 14 in July (S3b Fig), suggesting that we captured the majority of precipitation-derived CO_2_ contributions. Overall, we found that N-induced autotrophic consumption of CO_2_ was the primary control of CO_2_ fluxes at the daily-weekly timescales studied here. Future considerations of the connection between eutrophication and hard-water CO_2_ dynamics, however, cannot ignore the potential influence of CaCO_3_ precipitation, especially in lakes that are already prone to summer months with extreme pH (i.e., > 9–10; [2,13]).

## Conclusions

Hardwater lakes are geochemical hotspots that exchange CO_2_ with the atmosphere at rates far in excess of most aquatic ecosystems [1,13, this study]. Because these sites account for one-quarter of inland waters by area (~50% by volume), they play a quantitatively important role in the global CO_2_ cycle [1,2]. Although metabolic enhancement of CO_2_ influx increased rapidly after initial fertilization, the effects persisted for nearly a month, allowing sufficient time for chemical influences to interact with autotrophic processes to control atmospheric CO_2_ exchange. Importantly, strong effects of nutrient fertilization on CO_2_ flux were exhibited at even modest N amendment rates typical of both natural and non-point (diffuse) sources of N influx [45]. Given that N fertilization has increased exponentially since ca. 1960 [17–19], particularly with urea [26] in regions with decades of antecedent P amendment [20,23], we suspect many hardwater lakes now capture more CO_2_ than they did prior to the Anthropocene.

## Supporting information

S1 Fig Map of Wascana Lake and experiment location. a) continental location, b) gross drainage basin (1400 km^2^) and lake location, and c) depth contour map with the location of the mesocosm experiment (hatched area) and two long term monitoring sites (x).
(TIF)

S2 Fig Temporal patterns in mesocosm plankton dynamics. Effects of urea on (a) phytoplankton abundance (Chl *a*), (b) gross primary production (GPP), (c) bacterial density, (d) bacterial C consumption (BCC; production + respiration), (e) GPP:BCC, and (f) dissolved oxygen concentration (DO). Experimental enclosures received urea amendments of 0 (black circle), 1 (red circle), 3 (blue square), 8 (yellow diamond) and 18 mg N L^-1^ week^-1^ (grey triangle). Error bars = ± 1 S.E, and *n* = 3. Results of statistical analyses presented in S1 Table.
(TIF)

S3 Fig Temporal patterns in mesocosm inorganic carbon dynamics. Effects of urea on (a) pH, (b) dissolved inorganic carbon concentration (DIC), (c) partial pressure of CO_2_ in the water column (*p*CO_2_; dashed line indicates equilibrium with the atmosphere), and (d) net air-water CO_2_ flux (positive values above dashed line represent influx). Experimental enclosures received urea amendments of 0 (black circle), 1 (red circle), 3 (blue square), 8 (yellow diamond) and 18 mg N L^-1^ week^-1^ (grey triangle). Error bars = ± 1 S.E, and *n* = 3. Results of statistical analyses presented in S1 Table.
(TIF)

S4 Fig Temporal patterns in mesocosm limnological conditions. Limnological conditions during July, August, and September mesocosm experiments including concentrations of (a) total dissolved nitrogen (TDN), (b) total dissolved (TDP), (c) soluble reactive phosphorus (SRP), (d) dissolved organic carbon (DOC), (e) water transparency as Secchi disk depth, and (f) water temperature. Experimental enclosures received urea amendments of 0 (black circle), 1 (red circle), 3 (blue square), 8 (yellow diamond) and 18 mg N L^-1^ week^-1^ (grey triangle). Error bars = ± 1 S.E, and *n* = 3. Results of statistical analyses presented in S2 Table.
(TIF)

S1 Table Statistical results for temporal patterns in mesocosm plankton dynamics and C cycling. Repeated -measures analysis of variance (RM-ANOVA) of the effects of urea amendments (0, 1, 3, 8, or 18 mg N L^-1^ week^-1^) on variables related to biological production. Tukey’s HSD *post hoc* analyses indicate differences among treatments, and given probability levels (*P*) are presented for treatment and time by treatment effects. Statistics in bold indicate patterns significant at the *P* < 0.05 level. n.a. denotes occasions where samples were missing and analyses were not possible. All analyses used measurements from days 0, 7, 14, and 21 except July *p*CO_2_ and CO_2_ influx (days 0,7,14 only).
(DOCX)

S2 Table Statistical results for temporal patterns in mesocosm limnological conditions. Repeated-measures analysis of variance (RM-ANOVA) of the effects of urea amendment (0, 1, 3, 8, or 18 mg N L^-1^ week^-1^) on limnological conditions in mesocosms. Tukey’s HSD *post hoc* analyses indicate differences among treatments, and given probability levels (*P*) are presented for treatment and time by treatment effects. Statistics in bold indicate patterns significant at the *P* < 0.05 level.
(DOCX)

S1 Text Hypothetical mechanism by which addition of urea stimulates heterotrophic microbial production. (DOCX)

S1 Dataset Data underlying experimental analyses. (XLSX)
