# Supplementary figures and images for: Effects of experimental nitrogen fertilization on planktonic metabolism and CO2 flux in a hypereutrophic hardwater lake
Source: PLoS One. 2017 Dec 12;12(12):e0188652. doi: 10.1371/journal.pone.0188652 (PMC5726645; doi:10.1371/journal.pone.0188652)

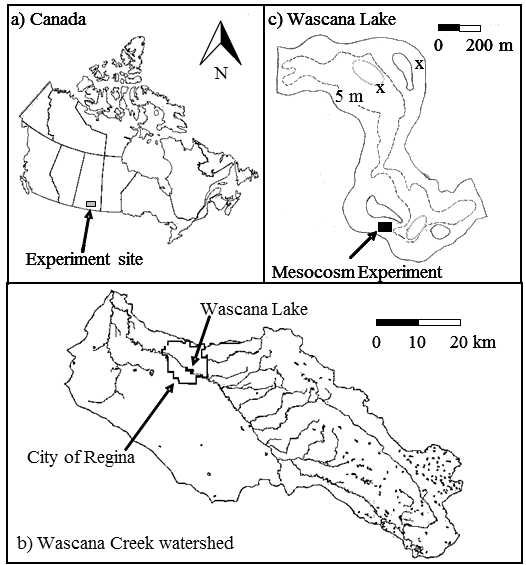

Supplement: S1 Fig — a) continental location, b) gross drainage basin (1400 km2) and lake location, and c) depth contour map with the location of the mesocosm experiment (hatched area) and two long term monitoring sites (x). (TIF) [file pone.0188652.s001.tif]

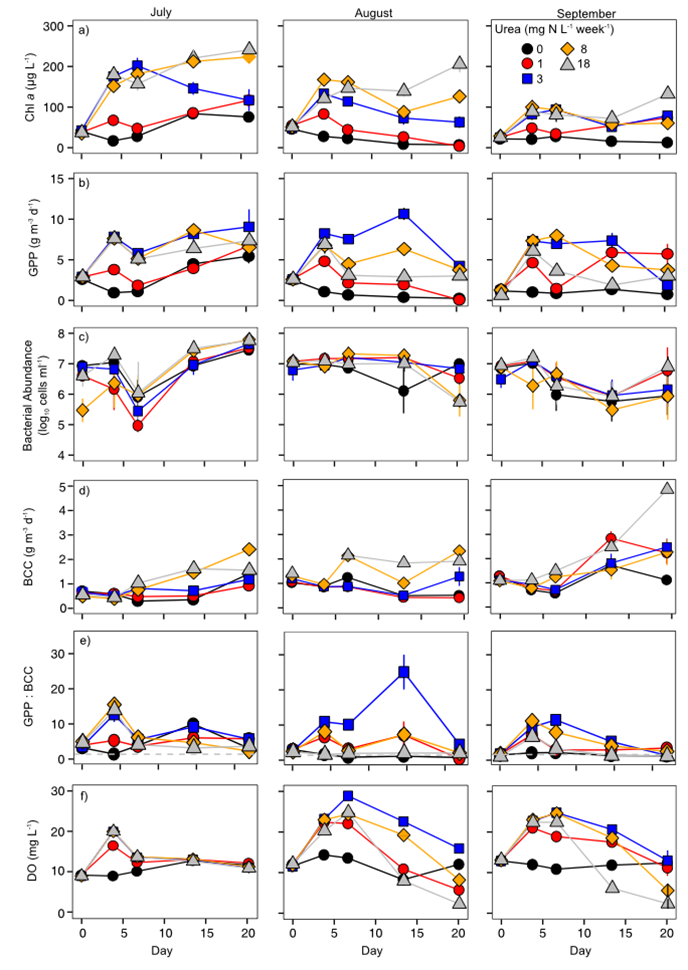

Supplement: S2 Fig — Effects of urea on (a) phytoplankton abundance (Chl a), (b) gross primary production (GPP), (c) bacterial density, (d) bacterial C consumption (BCC; production + respiration), (e) GPP:BCC, and (f) dissolved oxygen concentration (DO). Experimental enclosures received urea amendments of 0 (black circle), 1 (red circle), 3 (blue square), 8 (yellow diamond) and 18 mg N L-1 week-1 (grey triangle). Error bars = ± 1 S.E, and n = 3. Results of statistical analyses presented in S1 Table. (TIF) [file pone.0188652.s002.tif]

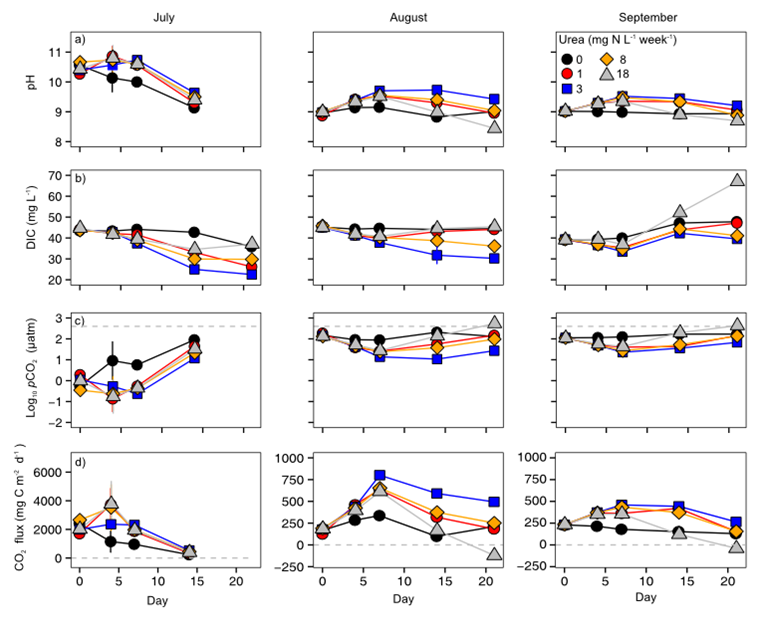

Supplement: S3 Fig — Effects of urea on (a) pH, (b) dissolved inorganic carbon concentration (DIC), (c) partial pressure of CO2 in the water column (pCO2; dashed line indicates equilibrium with the atmosphere), and (d) net air-water CO2 flux (positive values above dashed line represent influx). Experimental enclosures received urea amendments of 0 (black circle), 1 (red circle), 3 (blue square), 8 (yellow diamond) and 18 mg N L-1 week-1 (grey triangle). Error bars = ± 1 S.E, and n = 3. Results of statistical analyses presented in S1 Table. (TIF) [file pone.0188652.s003.tif]

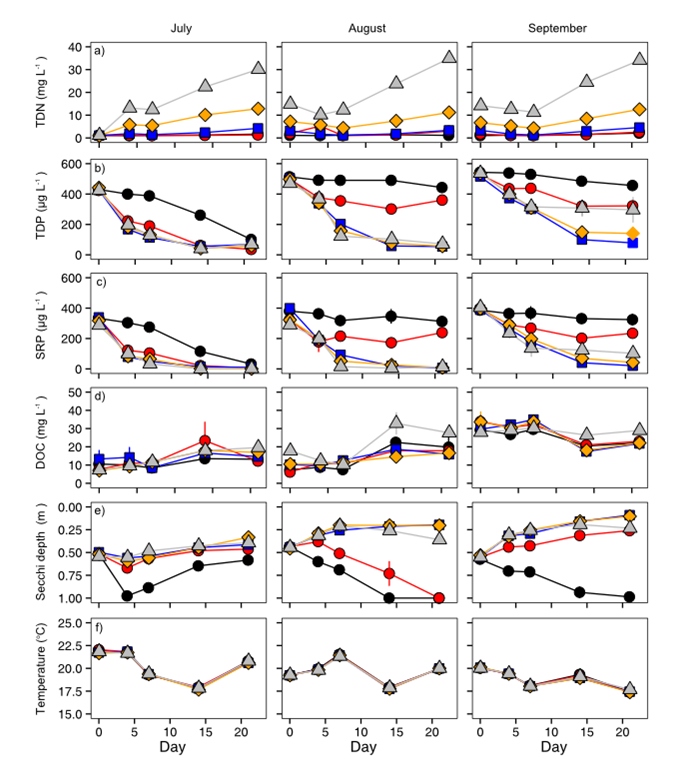

Supplement: S4 Fig — Limnological conditions during July, August, and September mesocosm experiments including concentrations of (a) total dissolved nitrogen (TDN), (b) total dissolved (TDP), (c) soluble reactive phosphorus (SRP), (d) dissolved organic carbon (DOC), (e) water transparency as Secchi disk depth, and (f) water temperature. Experimental enclosures received urea amendments of 0 (black circle), 1 (red circle), 3 (blue square), 8 (yellow diamond) and 18 mg N L-1 week-1 (grey triangle). Error bars = ± 1 S.E, and n = 3. Results of statistical analyses presented in S2 Table. (TIF) [file pone.0188652.s004.tif]
