# Supplementary material for: Effects of experimental nitrogen fertilization on planktonic metabolism and CO2 flux in a hypereutrophic hardwater lake
Source: PLoS One. 2017 Dec 12;12(12):e0188652. doi: 10.1371/journal.pone.0188652 (PMC5726645; doi:10.1371/journal.pone.0188652)
Supplement: S1 Table — Repeated -measures analysis of variance (RM-ANOVA) of the effects of urea amendments (0, 1, 3, 8, or 18 mg N L-1 week-1) on variables related to biological production. Tukey’s HSD post hoc analyses indicate differences among treatments, and given probability levels (P) are presented for treatment and time by treatment effects. Statistics in bold indicate patterns significant at the P < 0.05 level. n.a. denotes occasions where samples were missing and analyses were not possible. All analyses used measurements from days 0, 7, 14, and 21 except July pCO2 and CO2 influx (days 0,7,14 only). (DOCX) [file pone.0188652.s005.docx]

| Response Variable | July | |  | August | |  | September | |
| --- | --- | --- | --- | --- | --- | --- | --- | --- |
|  | *P* | *Post hoc* |  | *P* | *Post hoc* |  | *P* | *Post hoc* |
| Chlorophyll *a* biomass |  |  |  |  |  |  |  |  |
| Treatment | **< 0.001** | **18,8,3 > 3,1 > 1,0** |  | **< 0.001** | **18 > 8 > 3 > 1,0** |  | **< 0.001** | **18,3,8 > 3,8,1 > 0** |
| Interaction | **0.029** |  |  | **< 0.001** |  |  | **< 0.001** |  |
| Primary Production (GPP) |  |  |  |  |  |  |  |  |
| Treatment | **0.004** | **3,8,18 > 8,18,1 > 18,1,0** |  | **< 0.001** | **3 > 8 > 18 > 1 > 0** |  | **< 0.001** | **3,8,1 > 1,18 > 18,0** |
| Interaction | 0.08 |  |  | **< 0.001** |  |  | **< 0.001** |  |
| Bacterial density |  |  |  |  |  |  |  |  |
| Treatment | **0.004** | **18,8,3 > 3,1,0** |  | 0.44 | n.a. |  | 0.36 | n.a. |
| Interaction | **0.014** |  |  | 0.52 |  |  | 0.16 |  |
| Bacterial C consumption (BCC) |  |  |  |  |  |  |  |  |
| Treatment | **0.005** | **8,18,3 > 18,3,0 > 3,0,1** |  | **< 0.001** | **18,8 > 3,1,0** |  | **0.001** | **18 > 1,8,3,0** |
| Interaction | **< 0.001** |  |  | **< 0.001** |  |  | **< 0.001** |  |
| GPP : BCC |  |  |  |  |  |  |  |  |
| Treatment | **0.036** | **3,8,1,0 > 8,1,0,18** |  | **< 0.001** | **3 > 18,8,1,0** |  | **< 0.001** | **3,8 > 8,1 > 1,18,0** |
| Interaction | **0.005** |  |  | **0.003** |  |  | **< 0.001** |  |
| Dissolved O_2_ |  |  |  |  |  |  |  |  |
| Treatment | n.a. |  |  | **< 0.001** | **3 > 8 > 18,1 > 18,0** |  | **< 0.001** | **3,8,1 > 18,0** |
| Interaction | n.a. |  |  | **< 0.001** |  |  | **< 0.001** |  |
| pH |  |  |  |  |  |  |  |  |
| Treatment | **0.048** | **----** |  | **< 0.001** | **3 > 8,1 > 18,0** |  | **< 0.001** | **3,1,8 > 18,0** |
| Interaction | **0.010** |  |  | **< 0.001** |  |  | **< 0.001** |  |
| Dissolved inorganic carbon |  |  |  |  |  |  |  |  |
| Treatment | **0.002** | **0,18 > 18,8,1 > 8,1,3** |  | **< 0.001** | **0,18,1 > 18,1,8 > 3** |  | **< 0.001** | **18 > 0,1,8 > 1,8,3** |
| Interaction | **< 0.001** |  |  | **< 0.001** |  |  | **< 0.001** |  |
| *p*CO_2_ |  |  |  |  |  |  |  |  |
| Treatment | **0.006** | **0,1,18 > 1,3,8,18** |  | **0.008** | **0,1,18 > 1,3,8,18** |  | **< 0.001** | **18 > 0 > 1,3,8** |
| Interaction | **0.010** |  |  | **0.026** |  |  | **< 0.001** |  |
| Air-water CO_2_ influx |  |  |  |  |  |  |  |  |
| Treatment | 0.337 | **----** |  | **< 0.001** | **3 > 8,1 > 1,18 > 18,0** |  | **< 0.001** | **1,3,8 > 0 > 18** |
| Interaction | **0.013** |  |  | **< 0.001** |  |  | **< 0.001** |  |
|  |  |  |  |  |  |  |  |  |
