# Supplementary material for: Effects of experimental nitrogen fertilization on planktonic metabolism and CO2 flux in a hypereutrophic hardwater lake
Source: PLoS One. 2017 Dec 12;12(12):e0188652. doi: 10.1371/journal.pone.0188652 (PMC5726645; doi:10.1371/journal.pone.0188652)
Supplement: S2 Table — Repeated-measures analysis of variance (RM-ANOVA) of the effects of urea amendment (0, 1, 3, 8, or 18 mg N L-1 week-1) on limnological conditions in mesocosms. Tukey’s HSD post hoc analyses indicate differences among treatments, and given probability levels (P) are presented for treatment and time by treatment effects. Statistics in bold indicate patterns significant at the P < 0.05 level. (DOCX) [file pone.0188652.s006.docx]

| Response Variable | July | |  | August | |  | September | |
| --- | --- | --- | --- | --- | --- | --- | --- | --- |
|  | *P* | *Post hoc* |  | *P* | *Post hoc* |  | *P* | *Post hoc* |
| Total dissolved nitrogen |  |  |  |  |  |  |  |  |
| Treatment | **< 0.001** | **18 > 8 > 3,1,0** |  | **< 0.001** | **18 > 8 > 3,1,0** |  | **< 0.001** | **18 > 8 > 3,1,0** |
| Interaction | **< 0.001** |  |  | **< 0.001** |  |  | **< 0.001** |  |
| Total dissolved phosphorus |  |  |  |  |  |  |  |  |
| Treatment | **< 0.001** | **0 > 1,3,8,18** |  | **< 0.001** | **0 > 1 > 3,8,18** |  | **< 0.001** | **0,1 > 1,18 > 18,8 > 8,3** |
| Interaction | **< 0.001** |  |  | **< 0.001** |  |  | **< 0.001** |  |
| Soluble reactive phosphorus |  |  |  |  |  |  |  |  |
| Treatment | **< 0.001** | **0 > 1,3,8,18** |  | **< 0.001** | **0 > 1 > 3,8,18** |  | **< 0.001** | **0,1 > 1,18,8 > 18,8,3** |
| Interaction | **< 0.001** |  |  | **< 0.001** |  |  | **0.001** |  |
| Dissolved organic carbon |  |  |  |  |  |  |  |  |
| Treatment | 0.88 | ---- |  | **0.004** | **18 > 8,3,1,0** |  | 0.45 | ---- |
| Interaction | 0.51 |  |  | 0.27 |  |  | 0.08 |  |
| Secchi depth |  |  |  |  |  |  |  |  |
| Treatment | **< 0.001** | **0 > 1,3,8,18** |  | **< 0.001** | **0 > 1 > 3,8,18** |  | **< 0.001** | **0 > 1 > 18,3,8** |
| Interaction | **0.043** |  |  | **< 0.001** |  |  | **< 0.001** |  |
| Temperature |  |  |  |  |  |  |  |  |
| Treatment | 0.509 | ---- |  | 0.137 | ---- |  | 0.233 | ---- |
| Interaction | **0.034** |  |  | 0.158 |  |  | **0.034** |  |
|  |  |  |  |  |  |  |  |  |
|  | |  |  |  |  |  |  |  |
