# Supplementary material for: Effects of experimental nitrogen fertilization on planktonic metabolism and CO2 flux in a hypereutrophic hardwater lake
Source: PLoS One. 2017 Dec 12;12(12):e0188652. doi: 10.1371/journal.pone.0188652 (PMC5726645; doi:10.1371/journal.pone.0188652)
Supplement: S1 Text — (DOCX) [file pone.0188652.s007.docx]

**S1 Text.** **Hypothetical mechanism by which addition of urea stimulates heterotrophic microbial production.**

Enhanced heterotrophic growth and CO_2_ production in heavily-amended experiments (> 8 mg N L^-1^ week^-1^) (Fig 2d) may have arisen in part from the stimulation of nitrifying and denitrifying bacteria. Increased density of such bacteria have been recorded in previous mesocosms [1,2] and whole ecosystems fertilized with inorganic N [3,4]. Consistent with this mechanism, concentrations of NO_3_^-^  increased over time to account for 55.1 + 27.9% of TDN, whereas NH_4_^+^ was >10% of TDN only during the last week of trials with > 8 mg N L^-1^ week^-1^ (data not shown) despite the fact that urea decomposes directly to NH_4_^+^ [5]. In general, we hypothesize that microbial denitrification was not an important control of changes in inorganic N content in moderately fertilized mesocosms (i.e., <3 mg N L^-1^), as oxygen was often supersaturated throughout those trials (Fig 2f). Instead, denitrification may have contributed to N loss via atmospheric outgassing late in heavily amended experiments (> 8 mg N L^-1^), when waters were hypoxic (S2 Fig B1) and NO_3_^-^ was abundant. Together, these patterns suggest a more important role of chemolithotrophic bacteria in the transformation of dissolved N compounds and the regulation of water-column metabolism, although we recognize that further genetic, molecular, and enzymatic analyses are needed to better resolve changes in microbial community structure.

**References**

1. Sanderson MP, Bronk DA, Nejstgaard JC, Verity PG, Sazhin AF, Frischer ME. Phytoplankton and bacterial uptake of inorganic and organic nitrogen during an induced bloom of *Phaeocystis pouchetii*. Aquat Microb Ecol. 2008; 51: 153-168.
2. Finlay K, Patoine A, Donald DB, Bogard MJ, Leavitt PR. Experimental evidence that pollution with urea can degrade water quality in phosphorus-rich lakes of the Northern Great Plains. Limnol Oceanogr. 2010; 55: 1213-1230.
3. Barcia J, Kling H, Gibson J. Experimental manipulation of algal bloom composition by nitrogen addition. Can J Fish Aquat Sci. 1980; 37: 1175–1183.
4. Lathrop RC. Evaluation of whole-lake nitrogen-fertilization for controlling blue-green-algal blooms in a hypereutrophic lake. Can J Fish Aquat Sci. 1988; 45: 2061–2075.
5. Revilla M, Alexander J, Glibert PM. Urea analysis in coastal waters: Comparison of enzymatic and direct methods. Limnol Oceanogr Meth. 2005; 3: 290-299.
